# A Consolidation Curve Reproduction Based on Sigmoid Model: Evaluation and Statistical Assessment

**DOI:** 10.3390/ma15186188

**Published:** 2022-09-06

**Authors:** Bartłomiej Szczepan Olek

**Affiliations:** Department of Geotechnics and Strength of Materials, Cracow University of Technology, Warszawska 24, 31-155 Kraków, Poland; bartlomiej.olek@pk.edu.pl

**Keywords:** accuracy indices, clays, consolidation, inverse analysis, model performance statistics, parameter optimization, sigmoid

## Abstract

In the present study, various shapes of laboratory consolidation curves were numerically reproduced using a four-parametric sigmoid function. Sixteen consolidation curves were selected based on one-dimensional oedometer tests to statistically evaluate the sigmoid model and to determine the appropriate deviation statistics. Comparisons between observed and predicted data were performed using the following statistical metrics: mean error (*E*), root mean square error (*RMSE*), mean absolute error (*MAE*), weighted error (*WE*), revised Nash–Sutcliffe efficiency index (*CE*_1_) and refined index of model performance (*d_r_*). The weighted error (*WE*) was chosen as the optimization target in a first-order iterative optimization algorithm to determine a local minimum of a differentiable function. Comparing the simulated and observed settlements showed close correspondence in the values of *CE*_1_ and *d_r_* in terms of model performance. Based on statistical assessment, the maximum values of *RMSE* and *MAE* for the average degree of consolidation were 0.029 (-) and 0.021 (-), respectively. In turn the settlement data *RMSE* and *MAE* were 0.039 mm and 0.025 mm, respectively. These results indicated that the sigmoid expression effectively reproduced the shape of the consolidation curve.

## 1. Introduction

In physics and engineering applications, it is well known that a characteristic of a material or a system’s response to an external agent can be mathematically expressed in various forms. As stated by many researchers, a mathematical description of a material’s behaviour is helpful for numerical interpretation of material properties and for numerical analysis [1]. Many phenomena are evaluated based on collected laboratory data in geotechnical engineering practices. Due to the complexity of geotechnical issues and the heterogeneity properties of soils, the processes acting in soil mass are commonly described by the nonlinear curves. Nie and Guo [2] noted that a widely used method is to plot the nonlinear curve on a logarithmic axis to achieve an approximate straight line or several segments and model them by linear relationships. For instance, this kind of approach was successfully applied by Terzaghi and Peck [3], Butterfield and Baligh [4] and Liu et al. [5]. Regression analysis and curve fitting are ordinarily used to obtain the approximate analytic expression of discrete data. The curve-fitting technique is used to varying degrees in almost every geotechnical field. Curve fitting based on least squares has been extensively used to optimize parameters (e.g., [6,7,8,9,10,11,12,13,14,15,16,17] amongst many others) in view of its major importance in the context of interpretation of laboratory tests and evaluation field work. Curve fitting is the process of capturing a trend in data by assigning a single function across an entire range. In other words, a parameterised function that best matches a given set of data points is necessary. The main goal is to identify the coefficients of the parameterised function such that the approximate solution fits the data well. A parameterised function or a set of basic functions is selected considering data distribution to fit a given dataset to a curve. Then, a numerical method for least squares approximation is applied to minimise the sum of squared residuals of data points to determine the fitting coefficients. The curve-fitting technique is also part of an optimisation procedure with the aim of determining values for the model parameters that provide the best attainable fit between model predictions and corresponding observations [1]. Many parameterised functions have been proposed to describe geotechnical phenomena. Due to the multitude of phenomena occurring in the soil, in this article, we focus only on soil compressibility and rheology. One-dimensional compression of saturated soil subjected to an increment of total vertical stress consists of primary compression, which takes place during the dissipation of the excess pore water pressure, and a secondary compression, which follows at “virtually” constant effective vertical stress (no excess pore water pressure) [18]. The concept of primary and secondary compression has been extensively investigated through consolidation testing using an oedometer or hydraulically loaded Rowe cell. In general, two datasets can be obtained from a single laboratory test: compression data in terms of settlement of the sample and pore water pressure measured at the base of the consolidation cell. In routine practice, settlement of the sample is more often recorded than pore water pressure. Therefore, the present analysis is concerned only with a more favourable type of measurement. One of the main issues encountered when interpreting an oedometer test is the blurring of measurement data resulting from errors in reading the data and factors disturbing the readings, such as vibrations. In statistics and data processing for material science, smoothing a dataset involves creating an approximation function that to capture the main patterns in the data while ignoring noise or other small-scale phenomena. During smoothing, the data points of the signal are modified such that individual points higher than their neighbours (possibly due to noise) are reduced, and points lower than their neighbours are increased, leading to a smoother signal. Smoothing is often compared to curve fitting. Curve fitting usually involves the use of an explicit function form for the result. In contrast, the immediate results of smoothing are “smoothed” values without the subsequent use of a functional form if one exists. In the smoothed data, the residuals are not independent, and the distances of the smoothed points from the curve are not Gaussian. Therefore, the computed sum of squares will underestimate the true amount of scattering. Hence, the purpose of smoothing is to provide a general idea of relatively gradual changes in values, with little attention paid to the exact fit of the data values. In contrast, curve fitting focuses on achieving the closest possible fit.

The main purpose of this paper is to evaluate a mathematical model for prediction of time-settlement patterns based on a one-dimensional consolidation test. A review of stress–strain approximations for one-dimensional loading is presented in the next chapter, highlighting the difficulty of using one approximation type to recreate multiple consolidation curve shapes. Most of the functions discussed herein are best-suited for reproducing S-shaped curves. Therefore, they are unsuitable for curves characterized by a linear or non-linear segment of secondary compression. A potent and helpful tool is the Chapman–Richards growth model, which does not cope well with curves deviating from the S-shape, similarly to the FORE approach. In the present study, we assessed a four-parameter sigmoid model, with a focus on statistical analysis. In most works on parametric optimization of geotechnical models, the fit is evaluated based on the coefficient of determination (R^2^), which is not the optimal solution. The application of this parameter in practice is often misused and overinterpreted [19] or even misconceived in connection with the square of the correlation coefficient (*r*)^2^. With this in mind, the available statistical metrics were reviewed to determine the goodness of fit between observed and simulated results, and the most useful were selected. The collected data from oedometer tests performed on four soft organic soils were subjected to the minimization process by an iterative algorithm based on the steepest descent method. This type of soil was chosen due to its undesirable behaviour and irregular consolidation curve shapes. In most previous research, ideal S shapes of curves were reproduced. Therefore, in the present study, we selected curves with a variable curvature-to-linear shape and a rate that oscillates before stabilizing, which is a prime novelty. The following deviation statistics were used to assess the sigmoid model: mean error (*E*), root mean square error (*RMSE*), mean absolute error (*MAE*), weighted error (*WE*), revised Nash–Sutcliffe efficiency index (*CE*_1_) and refined index of model performance (*d_r_*).

## 2. Overview of Stress–Strain Approximations for One-Dimensional Loading

In engineering practice, compressibility and rheological phenomena in soft cohesive soils can be described using various mathematical functions designated for curve fitting. Ramberg and Osgood [20] described stress–strain curves of metals using a three-parameter function. The Ramberg–Osgood equation was also adapted for clays, although with a relatively poor fit [21]. The Chapman–Richards equation was derived by Richards [22] and Chapman [23] from the Von Bertalanffy growth equation. This equation is expressed as follows:

(1) $$y=\eta {\left[1-\kappa \;exp\left(-\mu t\right)\right]}^{\lambda }+\varepsilon$$

where *η*, *µ* and *λ* are constants; *κ* = *exp*(*µt*_0_); and *ε* = *y*_0_.

Nie and Guo [2] discussed the application of Chapman–Richards equation to many geotechnical problems. For example, this model expresses three types of nonlinear curves, i.e., the degree of consolidation (*U_v_*) versus time factor (*T_v_*) curves in one-dimensional consolidation, plane strain consolidation under strip loading and the compressibility and permeability curves of soft clay. Nie and Guo [2] also pointed out that the Gompertz, Mitscherlich and logistic growth equations are special cases of the Chapman–Richards function. The equations for Gompertz, Mitscherlich and logistic growth can be respectively expressed as:

(2) $$y=\frac{A^{\prime }}{1+exp\left(a-b^{\prime }t\right)}$$

(3) $$y=A^{\prime }\left[1-exp\left(-b^{\prime }t\right)\right]$$

(4) $$y=A^{\prime }exp\left[-exp\left(a^{\prime }-b^{\prime }t\right)\right]$$

where *A*′, *a*′ and *b*′are experimentally determined parameters.

Many results of structural and mechanical tests demonstrate a hyperbolic character. An equation for a rectangular (equilateral) hyperbola was proposed by Ayrton and Perry [24] and later developed by Southwell [25] to explain the behaviour of eccentrically loaded thin elastic struts. Such test results form a curve that passes through the origin and tends toward limiting values in much the same manner as a rectangular hyperbola with horizontal and vertical asymptotes. Duncan and Chang [26] used a hyperbolic function to describe load-settlement behaviour of piles. Chin [27], Tan [28], and Sridharan and Rao [29] observed the hyperbolic relationship between settlement and time. Sridharan and Rao also used a hyperbolic approach to derive the coefficient of consolidation (*c_v_*) and the end of primary consolidation point (EOP) from laboratory data. Handy [16] adopted the first-order rate equation (a special case of the Mitscherlich growth model) to specify the rate of a physical process in geotechnical engineering when the equilibrium condition is approached. The geotechnical response to be modelled decreases with time (*t*) in an exponential or log-linear manner. Among many considered data from various tests, Handy used FORE to reproduce primary consolidation as follows:

(5) $$log\left(e-e_{p}\right)=-\left(k_{t}t+C_{t}\right)$$

where *e* is the void ratio, *e_p_* is the final void ratio, *t* is the consolidation time, and *k_t_* and *C_t_* are experimentally determined constants.

The viscous behaviour of soft clays is usually caused by a time-dependent creep process. Le and Khabbaz [30] distinguished five groups of factors that cause soil creep, namely (a) the breakdown of interparticle bonds, (b) sliding between soil particles, (c) water flow from micropores to macropores, (d) deformation due to structural viscosity and (e) deformation due to the jumping of bonds. Creep deformation can be defined in several ways, the simplest of which concerns the destruction or adjustment of soil structure under constant effective stress. Comprehensive elaborations on this time-dependent phenomenon were provided by Lingaard [31], Leroueil [32], and Kaczmarek and Dobak [33]. Many formulations for soil creep have been proposed over the years; selected examples are presented below. Buisman [34] suggested a logarithmic equation for the creep deformation in the following form:

(6) $$\varepsilon_{s}\left(t\right)=L\cdot ln\left(\lambda_{0}\cdot t+1\right)$$

where *L* and *λ*_0_ are experimentally determined parameters, and *t* is the time.

Singh and Mitchell [35] proposed a three-parameter model to describe the creep behaviour of soils, adopting exponential and power functions. Havel [36] evaluated various formulations for primary and secondary compression using Sigma Plot 8.0 software. In the case of approximation of strain–time curves, exponential, logarithmic (Buisman equation) and sigmoid functions were considered. In the case of secondary consolidation, Havel incorporated the exponential equation originally proposed by Kohlrausch [37]:

(7) $$\varepsilon_{s}\left(t\right)=C_{0}\left[1-\exp\left(-\Delta_{0}\cdot t^{x_{0}}\right)\right]$$

where *C*_0_ and *x*_0_ are experimentally determined parameters, and *t* is the time.

Kohlrausch also evaluated several sigmoid functions. The following approximations were assessed for primary and secondary consolidation:

(8) $$\varepsilon_{p+s}\left(t\right)=y_{p0}+\frac{a_{p}}{1+{\left(\frac{t}{x_{po}}\right)}^{b}}$$

where *y_po_*, *a_p_*, *x_po_* and *b* are experimentally determined parameters.

(9) $$\varepsilon_{s}\left(t\right)=y_{s0}+a_{p}\cdot \left(1-exp\left(-{\left(\frac{t-x_{p}+b_{p}\cdot ln2^{\frac{1}{c_{p}}}}{b_{p}}\right)}^{c_{p}}\right)\right)$$

where *y_so_*, *a_p_*, *x_p_*, *b_p_* and *c_p_* are experimentally determined parameters.

(10) $$\varepsilon_{p+s}\left(t\right)=y_{p0}+\frac{a_{p}{}^{t^{b_{p}}}}{\left(c_{p}^{b_{p}}+t^{b_{p}}\right)}$$

where *y_po_*, *a_p_*, *c_p_* and *b_p_* are experimentally determined parameters.

Equations (8)–(10) are suitable to approximate data in which a small oscillation of the independent variable (for example, vertical strain or compression) causes a large oscillation of the dependent variable (for example, Janbu’s time resistance (*R*) or settlement potential (*S*) [38]). The examples quoted here illustrate the multitude of forms of mathematical expressions that can approximate a consolidation curve. In the majority of these cases, the consolidation curve exhibits S-shaped or quasi-S-shaped behaviour using a semi-logarithmic diagram. However, experimental evidence reveals varied shapes of consolidation curves. A geometric shape of a consolidation curve depends on relative magnitudes of primary and secondary compression. In particular, the geometric shapes vary depending on the soil type and test conditions, which are commonly expressed by the load increment ratio (LIR = ∆*p*/*p*) [39]. The load increment ratio signifies the change in consolidation stress divided by the initial consolidation stress. Based on the classification proposed by Marsal et al. [40] and further repeated by Leonards and Girault [41], three types of consolidation curves can be obtained in a consolidation test. Subsequent studies conducted for various types of soil confirmed previous observations [42,43,44,45]. In summary, the time rate of consolidation is dependent on the LIR used during the test. With large LIRs, a consolidation curve is obtained that, with exception of secondary compressions, is characterized by the Terzaghi theory (type I S-shaped curve). With small LIRs, a type III curve is obtained, whereas with intermediate LIRs, a transition curve (type II) is observed. Type II and type III curves are also characteristic of a load increment that straddles the effective preconsolidation pressure [41]. These curves are strongly nonlinear, and their shapes vary with time. Zhang [46] proposed a sigmoid-type expression for rate of consolidation and settlement predictions. The fitted curve has an expression with four fitting coefficients of the sigmoid as follows:

(11) $$y\left(t\right)=a_{2}+\frac{a_{1}-a_{2}}{1+{\left(\frac{t}{x_{0}}\right)}^{n}}$$

where *a*_1_, *a*_2_, *x*_0_ and n are experimentally determined parameters.

In the adopted model, parameter *a_1_* controls the position of the curve, parameter *a_2_* controls the slope of the last section of the curve (responsible for the slope of the part describing the secondary consolidation) and the other parameters are responsible for the curvature. A closer examination of Equation (11) suggests that this kind of sigmoid function would be suitable to reproduce various shapes of consolidation curves. Therefore, the present study is concerned with this expression. Other expressions mentioned above were not included in the present analysis due to limitations indicated by the authors who proposed them.

## 3. Theoretical Background

The consolidation phenomenon links the hydraulic and the mechanical behaviour of saturated soils to model the dissipation of excess pore water pressure resulting from compression. Given that any change in pore water pressure is equal to the change in effective stress, for a homogeneous soil under constant applied total stress, the continuity equation for one-dimensional consolidation can be expressed as [47]:

(12) $$\left(\frac{1}{1+e_{0}}\right)\frac{\partial e}{\partial t}=-\frac{k}{\gamma_{w}}\frac{\partial^{2}\sigma^{\prime }}{\partial z^{2}}$$

where *k* is the coefficient of permeability, *σ’* is the effective stress, *e*_0_ is the initial void ratio, e is the void ratio at time *t* and *γ_w_* is the unit weight of water.

Including the relationship between the coefficient of volume compressibility (*m_v_*), void ratio (*e*) and the effective stress from the previous compound (see Equation (12)), the following expression is obtained:

(13) $$-m_{v}\frac{\partial \sigma^{\prime }}{\partial t}=-\frac{k}{\gamma_{w}}\frac{\partial^{2}\sigma^{\prime }}{\partial z^{2}}$$

Finally, each increase in the effective stress consequently causes the simultaneous dissipation of the pore water pressure, which can be expressed as follows:

(14) $$\frac{\partial u}{\partial t}=\frac{k}{m_{v}\gamma_{w}}\frac{\partial^{2}u}{\partial z^{2}}$$

For convenience, the first term of the right side of Equation (14) is included in one factor, i.e., the coefficient of consolidation (*c_v_*). Then, a one-dimensional partial differential equation that governs the consolidation process and the dissipation of excess pore water pressure is expressed as:

(15) $$\frac{\partial u}{\partial t}=c_{v}\frac{\partial^{2}u}{\partial z^{2}}$$

where *u* is excess pore water pressure, and *c_v_* is the coefficient of consolidation.

For the full mathematical derivation of the consolidation equation, the reader is referred to Terzaghi and Peck [48]. The closed-form expression of the average degree of consolidation (*U_v_*) in terms of the time factor (*T_v_*) is expressed as [48]:

(16) $$U_{v}=1-\frac{4}{\pi^{2}}\sum_{n=0}^{\infty }\frac{2}{{\left(2n+1\right)}^{2}}exp\left[-{\left(\frac{2n+1}{2}\pi \right)}^{2}T_{v}\right]$$

where *n* is an integer, and *T_v_* is the dimensionless time factor (*T_v_* = *c_v_t/H*^2^, where *c_v_* is the coefficient of consolidation, H is the vertical drainage path and t is the consolidation time).

The average degree of consolidation (*U_v_*) is a global measure of the process equal to the percentage consolidation settlement. The experimental degree of consolidation calculated based on observed deformation or changes in the sample height can be expressed as:

(17) $$U_{v\left(\mathrm{experimental}\right)}=\frac{h_{0}-h_{i}}{h_{0}-h_{f}}$$

where *h*_0_ is an initial sample height under the analysed stress level, *h_i_* is the sample height at the analysed time (*t*) and *h_f_* is the ultimate sample height at the end of consolidation under the given value of load increment.

## 4. Materials and Methods

### 4.1. Soil Material Used in the Present Study

In order to verify the usefulness of the considered sigmoid function for the approximation of the consolidation curve, a series of consolidation tests were executed on soft organic soils from Żuławy Fens. The choice was dictated by the intention to obtain settlement–time curves with varied shaped. Organic clays and silts were specifically chosen as soils that exhibit considerable creep deformation with settlement–time curve shapes that significantly differ from ideal S shapes. The physical parameters of soils used in the present study are given in Table 1. Despite different intrinsic properties (plastic and liquid limits) and plasticity index ranges, the O1–O3 soil consolidation curves showed a similar course, mainly because these soils comprise a mixture of similar fractions with different proportions and have the same geological origin, and the LIR controls their ratio of primary to secondary compression, resulting in similar creep susceptibility. The O4 soil exhibited distinct consolidation behaviour, with deformations caused by creep from the beginning of loading, independent of the applied LIR.

### 4.2. Testing Procedure

**Figure 1:**
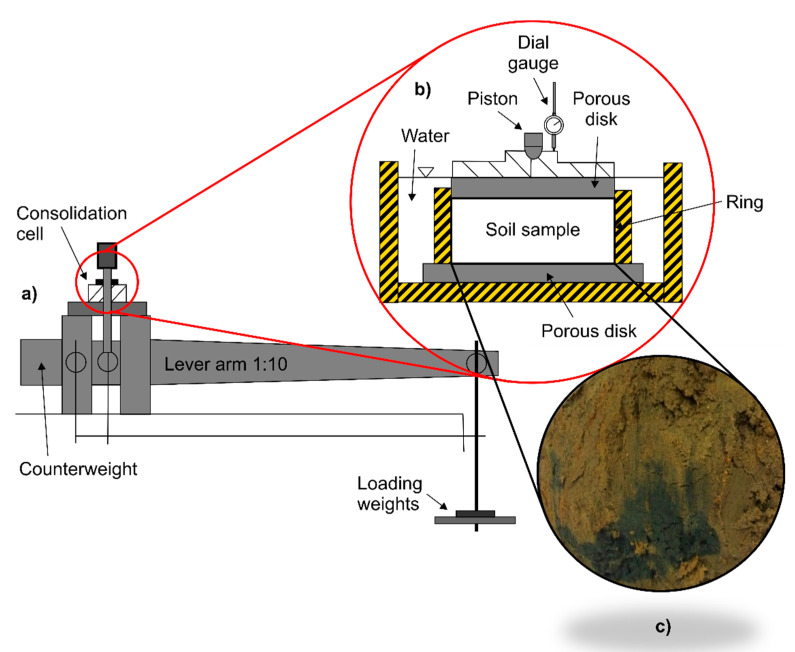
Arrangement of oedometer testing equipment: (**a**) schematic diagram of the oedometer apparatus; (**b**) schematic of the consolidation cell; (**c**) example of the organic soil material used in the present investigation (grey-to-black streaks denote decomposed humus in the brown loamy silt matrix).

Laboratory experiments were conducted according to ISO/TS 17892-5 [49] using a conventional incremental loading oedometer with a ring size of 60 mm diameter and 20 mm thickness (see Figure 1). In the oedometer test, the cylindrical soil sample is laterally restrained by a stainless ring, and the top and bottom faces of the specimen are contacted with porous discs. Thus, specimen drainage takes place in the vertical direction, and the consolidation is one-dimensional. According to the testing procedure, the soil sample is kept under water during the test, and the load is applied through a lever arm. In the study presented herein, each load was kept for 5 days to precisely capture secondary consolidation. The oedometer tests were performed by changing the LIR value. In this case, the following loading scheme was implemented: 25 kPa → 50 kPa → 150 kPa → 250 kPa. This procedure resulted in obtaining varied geometric types of consolidation curves. For each load increment, the specimen deformation expressed as relative compression (settlement) and the corresponding time were noted.

**Figure 2:**
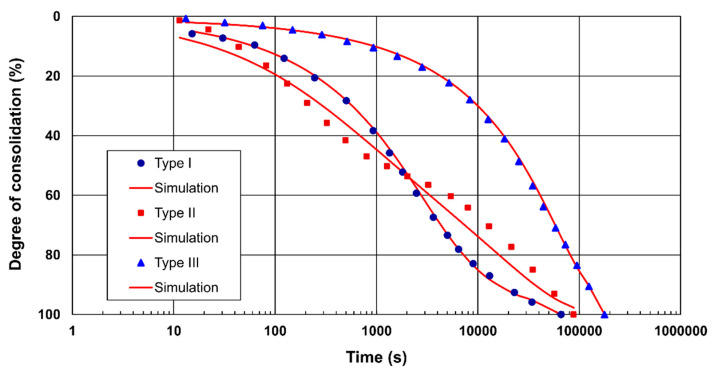
Types of consolidation curves: type I—classic S-shaped (convex-to-concave shape), type II—variable curvature-to-linear shape with a rate that oscillates before stabilizing, type III—concave-to-linear shape with an initially monotonous decrease followed by stabilization.

In this work, a two-stage verification of the proposed equation for consolidation curve approximation and a comparative procedure validation were carried out. In the first stage of the analysis, possible types of consolidation curves were studied due to their geometric shape. Basic types of consolidation curves are presented on Figure 2. The original data collected by Marsal et al. [40] were selected and generated using Plot Digitizer software [50]. The data presented in Figure 2 were normalised using a definition of average degree of consolidation (*U_v_*). Hence, the geometric shape of the consolidation curves is preserved regardless of the amount of compression for a given increase in the load controlled by the LIR. The average degree of consolidation is directly proportional to the percentage consolidation and describes an advancement of the consolidation process. Due to the lack of available values for the initial sample height (*H*_0_) and complete records of all load steps in the studies by Marsal et al., the standard value of the initial sample height (*H*_0_ = 20 mm) was adopted for each curve. This assumption did not change the shape of the consolidation curves.

In order to identify the sigmoid model parameters (*a*_1_, *a*_2_, *x*_0_ and *n*) for each consolidation curve, we used a procedure developed by Olek [51] based on the first-order iterative algorithm to determine the minimum of a function necessary to solve the inverse problem. During the inverse analysis, a given model is calibrated by iteratively changing input values until the simulated output values match the observed data. Initial value ranges of parameters for calibration were set during the parameterization stage, whereas fitting parameter values were involved in the calibration stage. To determine the optimal set of parameters to minimize the difference between the experimental data and the prediction results, a function that evaluates the discrepancy between the model prediction and the experimental data was defined. Then, regression analysis was performed to minimize this function. The weighted error (*WE*) was chosen as the optimization target to determine a function’s local minimum. *WE* specifies the average difference between the observed and simulated results, expressed in the normalized form of the least squares method [52]. Further steps of the adopted procedure assumed: the calculation of the remaining statistical indices, converting the average degree of consolidation (*U_v_*) into the settlement data and reconstructing the consolidation curve in the time-settlement space.

### 4.3. Statistical Analysis

In this section, we will describe each evaluation metric used to describe the performance and efficiency of the sigmoid model (Equation (11)). In the following equations, *x_i_* is the observed (i.e., independent variable) value, *y_i_* is the predicted (i.e., dependent variable) value, $\bar{x}$ is the mean of the observed data and $\tilde{y}$ is the mean of the predicted values. In regression analysis, the coefficient of determination (or squared multiple correlation coefficient) (*R*^2^) is commonly used to determine the appropriateness of the fitted model in explaining the variations in a given dataset:

(18) $$R^{2}={\left[\frac{\sum_{i=1}^{n}\left(x_{i}-\bar{x}\right)\left(y_{i}-\tilde{y}\right)}{\sqrt{\sum_{i=1}^{n}{\left(x_{i}-\bar{x}\right)}^{2}\sum_{i=1}^{n}{\left(y_{i}-\tilde{y}\right)}^{2}}}\right]}^{2}$$

where *i* is the *i*th value (e.g., 1st, 2nd…), *n* is the number of observations and Σ is the summation symbol.

*R*² is a measure of how strongly two variables are correlated and provides a summary measure for the goodness of fit of any linear regression model. It is based on the proportion of the variability of the study variable that can be explained through the knowledge of a given set of explanatory variables [53]. Despite the suggestive interpretation of this metric, it can easily be overinterpreted and does not provide sufficient information to determine the validity of the correlation [54,55]. The limitations of correlation-based statistics can be overcome by using deviation statistics. Deviation is a measure of difference between the observed and predicted values, i.e., *d* = *y* − *x*. The most common efficiency measure is mean error (*E*) [56]:

(19) $$E=\sum \left(y_{i}-x_{i}\right)/n$$

Mean error (*E*) indicates whether the model-simulated y tends to overestimate (*E* > 0) or underestimate (*E* < 0) the observed *x* data [57]. Whereas positive and negative errors can negate one another, the mean error is subject to a severe drawback in validation analysis. The statistical parameters of residual error between observed and predicted datasets are divided into absolute and relative parameters. Due to the verification of one model concerning the experimental data, only absolute measures were used in this work.

The commonly used absolute parameters to provide a quantitative assessment of model error expressed in terms of the same units as the original data are root mean square error (*RMSE*) and mean absolute error (*MAE*). *RMSE* is the standard deviation of the residuals:

(20) $$RMSE=\sqrt{\sum_{i=1}^{n}{\left(x_{i}-y_{i}\right)}^{2}/n}$$

Residuals measure the distance of data point from the regression line. The main difference between *RMSE* and *MAE* is that changes in *MAE* are linear, with scores measured as the average of the absolute error values. *MAE* evaluates all deviations from the observed values, regardless of sign:

(21) $$MAE=\sum_{i=1}^{n}\left|x_{i}-y_{i}\right|/n$$

Both *RMSE* and *MAE* range from 0 to ∞ and are negatively oriented scores, which means lower values are preferrable. In order to reduce factors that can influence error measure, weighted metrics can be used [58]. Therefore, factors such as the shape of the experimental curve, the number of measurement points and the scale effects on the fitness between the observed and the predicted results are reduced by adopting weighted metrics for each calculation point:

(22) $$WE=\frac{\sum \frac{\left|x_{i}-y_{i}\right|}{x_{i}}}{\sum \left(\frac{y_{i}-y_{i-1}}{2}\right)+\left(\frac{y_{i+1}-y_{i}}{2}\right)\;}$$

Dimensionless coefficients that contrast model performance with accepted norms or standards constitute a separate group of metrics. The essential representative of this family of parameters is the previously introduced *R*^2^. Another statistic used to evaluate fitness is the coefficient of efficiency (*CE*) [59], also known as the Nash–Sutcliffe efficiency index (*E_f_*) [60]. This parameter determines the relative magnitude of the residual variance compared to the measured data variance [61]. *CE* can be modified by replacing the sum of squares term with the sum of absolute values of *y* − *x* [62]. Both measures are dimensionless and range from −∞ to 1.0 (*CE* or *CE*_1_ = 1 corresponds to a perfect match of modelled output with the observed data):

(23) $$CE_{1}=1-\frac{\sum_{i=1}^{n}\left|x_{i}-y_{i}\right|}{\sum_{i=1}^{n}\left|x_{i}-\bar{x_{i}}\right|}$$

The refined index of model performance (*d_r_*) is a statistical measure of model performance related to increases and decreases in *MAE* [63]:

(24) $$d_{r}=1-\frac{\sum_{i=1}^{n}\left|x_{i}-y_{i}\right|}{2\sum_{i=1}^{n}\left|x_{i}-\bar{x_{i}}\right|}\;$$

where *d_r_* is dimensionless and varies from −1.0 to 1.0 (*d_r_* = 1 corresponds to a perfect match of modelled output with the observed data).

## 5. Results

### 5.1. Marsal et al. Cases

In the first stage of evaluation of the applied sigmoid model, data from Marsal et al. were used (see Figure 2). Based on the lowest values of *WE*, which is the target for the optimization in the present study, a set of model parameters were ultimately adopted. *R*^2^, *E*, *RMSE*, *MAE*, *WE*, *CE*_1_ and *d_r_* were computed using Equations (18)–(24) and are presented in Table 2. In the case of type I and III curves, an excellent representation of the observed data was obtained, as confirmed by the calculated values of the deviation statistics (Table 2). *RMSE*, *MAE* and *CE*_1_ should not be used alone. Applying these indicators together allows for the creation of a set of model selection criteria that offsets the individual limitations of each indicator. A comparison of *RMSE* and *MAE* deviation statistics obtained for three types of curves reveals that their values for type II curves are higher than those for type I and III curves because type II curves are burdened with the most significant uncertainty related to the behaviour of the soil i.e., overlapping primary and secondary consolidation and substantial changes in the structure of the soil skeleton and pore space. The worst response of the model was obtained for the type II curve, for which dimensionless measures, such as *CE*_1_ and *d_r_*, had the lowest values. *CE*_1_ and *d_r_* are scaled algebraic descriptions of average error magnitude. An advantage of *d_r_* over *EF*_1_ is its wider overall range to adequately resolve the variety of ways that predicted values could differ from observed values. In turn, d_r_ has a structure similar to that of *EF*_1_ but with a substantially different scaling and lower limit [49]. Due to their unusual shape, type II curves are difficult to reproduce using only one approximating function. Despite the relatively high values of *E*, *RMSE* and *WE* in this example, the assessed sigmoid model was not clearly excluded at this stage of the analysis. One possible solution for improved approximation is to split this curve into two segments and model each separately.

### 5.2. Soft Organic Soils

Sixteen consolidation curves were selected based on the one-dimensional oedometer tests to statistically evaluate Equation (11) and to determine the appropriate deviation statistics (*E*, *RMSE*, *MAE*, *WE*, *CE*_1_ and *d_r_*). Both graphical and statistical comparisons were considered. The time-settlement datasets from the above curves had various separate observations depending on the frequency of the measurements for each load increment during consolidation. Each time-settlement dataset was converted into a time degree of consolidation dataset for comparison purposes.

**Figure 3:**
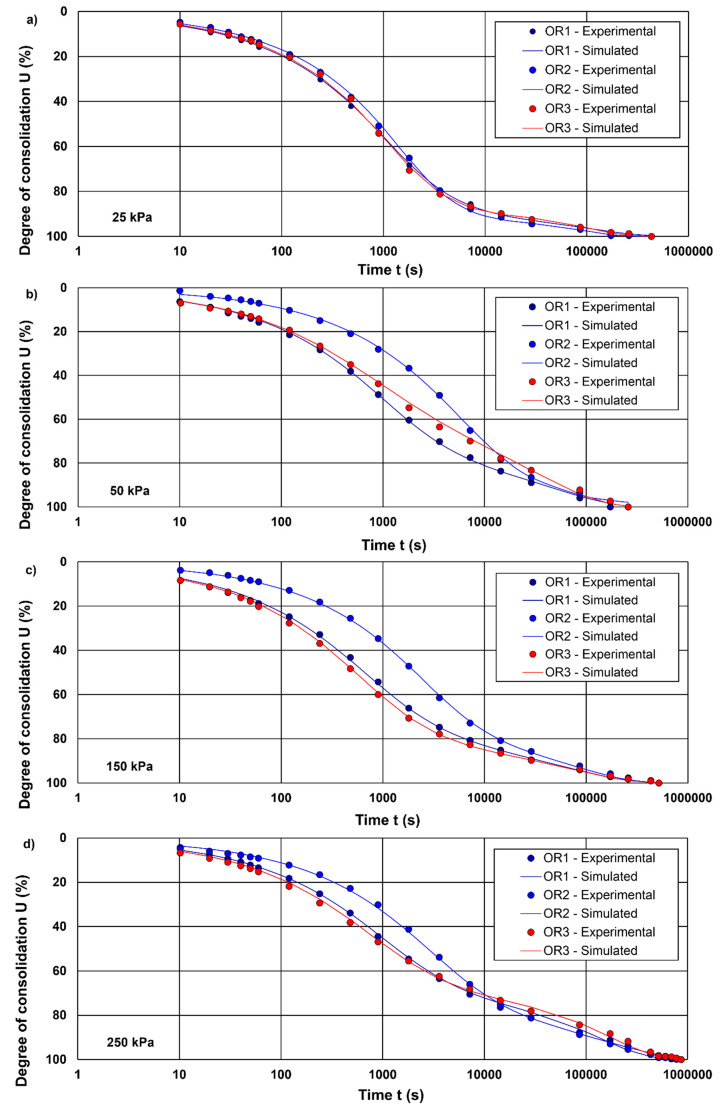
Comparisons between simulations and experiments for soft organic soils: (**a**) LIR = 1.0; (**b**) LIR = 0.5; (**c**) LIR = 0.66; (**d**) LIR = 0.4.

**Figure 4:**
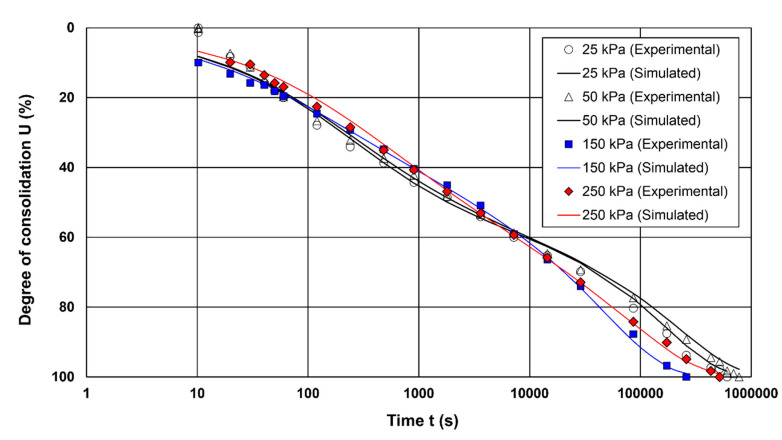
Comparisons between simulations and experiments for soft organic soil O4 exhibiting large deformations due to secondary consolidation.

Figure 3 and Figure 4 presents a comparison of simulated and observed curves obtained in the tests. In this study, four cases were considered: case I (25 kPa; LIR = 1), case 2 (50 kPa; LIR = 0.5), case III (150 kPa; LIR = 0.66) and case IV (250 kPa; LIR = 0.4). Therefore, each case is based on a different consolidation pressure and/or load increment ratio. The optimal parameters and the deviation statistics are presented in Table 3. In general, the laboratory investigation produced two types of settlement-time patterns, i.e., type I and II curves. As shown in Figure 3, cases I, II and III are associated with type I curves, whereas case IV is related to type II curve. Both types of curves can be characterized by one or more segments of secondary compression (the latter part of the consolidation curve). The main difference is a variable rate of secondary compression associated with type II curves. A visual inspection of the simulated and observed consolidation curves shows that the sigmoid model is reasonable. However, the actual observations are overestimated or underestimated in all the simulated data cases (see Figure 3a–d), although the simulations preserved the trend of the measurements, which is why the *R*^2^ value was close to 1.0. Although simulation considerably overestimated and underestimated the measurements in the case of type II curves, Marsal’s original data are characterised by a high *R*^2^ value, e.g., *R*^2^ = 0.98. Consequently, this metric is not adequate to evaluate the quality of the simulations.

Model overestimation or underestimation of measurements is assessed by the mean error (*E*). Despite the clear meaning of this index, unambiguous evaluation is difficult. This vague assessment may be due to the fact that some segments of the consolidation curve were overestimated and others were underestimated in various proportions. For most of the analysed data, *E* indicates that the simulated data only marginally overestimated the measurements. The highest value of *E* was reported for sample O4 for a consolidation pressure of 50 kPa (i.e., LIR = 0.5). In the remaining cases, such as sample O1 (*σ* = 50; LIR = 0.5), sample O2 (*σ* = 250; LIR = 0.4), sample O3 (*σ* = 50; LIR = 0.5) and sample O4 (*σ* = 250; LIR = 0.4), simulations slightly underestimated the measurements.

**Figure 5:**
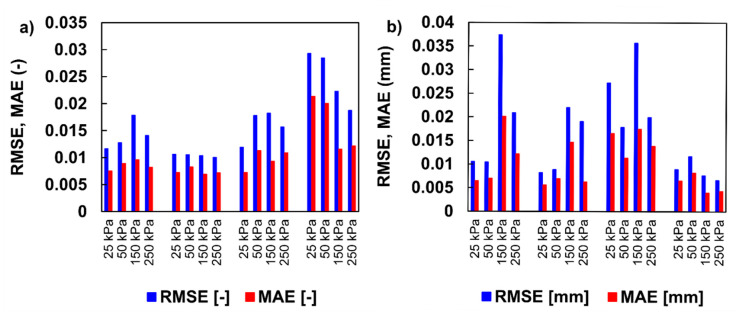
Diagrams of RMSE and MAE for two types of consolidation data: (**a**) average degree of consolidation; (**b**) settlement.

Figure 5 presents diagrams of *RMSE* and *MAE* under different consolidation pressures. Overall, the figure demonstrates that for two types of data, i.e., average degree of consolidation *U_v_* (-) and settlement (mm), *RMSE* values were higher than those of *MAE*, which is the common case [61]. The maximum values of *RMSE* and *MAE* for *U_v_* were 0.029 (-) and 0.021 (-), respectively. For the settlement data, these criteria reached values of 0.039 mm and 0.025 mm, respectively. In this sense, the small deviations indicate that the predicted model is close to the real value. 

A small deviation of those metrics (*RMSE* = 0.017 mm, on average, and *MAE* = 0.010 mm, on average, for all simulations) was found between the simulated settlement and the observed data, indicating that Equation (11) can correctly reproduce the shape of the consolidation curve. *RMSE* or *MAE* do not provide about the level or degree of error; therefore, they should be linked to other statistical metrics. The poorest fitness between the simulated and observed data assessed by *RMSE* and *MAE* was obtained for O4 soil under a load of 25 and 50 kPa (i.e., LIR = 1 and LIR = 0.5, respectively) for compression developed in the first few seconds after the load was applied. This could be attributed to uncertainties related to soil response immediately after load application, i.e., instant compression resulting from the dramatic changes in the soil structure in the initial phase of the test. With respect to the initial state of the soil (structured/unstructured) resulting from the sedimentation processes during which the soil was formed, the structural sensitivity of the material and disturbations generated during sampling are also critical.

Weighted error (*WE*) is a type of scalar error function used to characterize the discrepancy between experimental behaviour and predicted behaviour. In this work, *WE* was selected as the optimization target (minimization) in the employed gradient-based method to determine the optimal model parameters. In this respect, other dimensionless metrics, such as *EF*_1_ and *d_r_*, were calculated for an optimised set of parameters according to *WE*. The *WE* metric was linked to the experimental uncertainty of the measurement point by adjusting the weights of measured points. The calculated values of *WE* ranged from 0.015 to 0.061, clearly indicating that reasonable agreement between simulated and experimental data was achieved. In the case of sample O4 (*σ* = 25 kPa; LIR = 1 and *σ* = 50 kPa; LIR = 0.5), a type II consolidation curve was obtained, and the simulated results were characterised by the highest *WE* values (0.061 and 0.056, respectively). The values of *MAE* were also the highest for these two curves among all considered simulations. Based on the data presented in Table 3, linear regression was obtained between these two statistics with *R*^2^ = 0.86 (*y* = 0.2992*x* + 0.0025), which clearly demonstrates that *WE* increases with increased *MAE* values.

The lowest *WE* values were associated with S-shaped curves (type I). In addition to *WE*, single-value indices, such *CE*_1_ and *d_r_*, were also used as a goodness-of-fit statistics. The ranges of *CE*_1_ and *d_r_* were 0.989 to 0.920 and 0.998 to 0.976, respectively, showing that *CE*_1_ had a relatively wider range than *d_r_*, whereas *d_r_* is less sensitive to deviation.

Criteria for the coefficient of efficiency (*CE*) were developed based on an investigation reported by Ladson [64]. Consequently, *CE* values can be categorized as follows: *CE* ≥ 0.93, excellent; 0.8 ≤ *CE* < 0.93, good; 0.7 ≤ *CE* < 0.8, satisfactory; 0.6 ≤ *CE* < 0.7, passable; and *CE* < 0.6, poor. Other model performances based on the coefficient of efficiency are reported in the literature [48]; however, those reported by Ladson are more conservative. Therefore, due to a lack of criteria for *CE*_1_, the information provided by Ladson might be helpful. All except one simulation (sample O4: *σ* = 150 kPa; LIR = 0.66) evaluated in the present study could be regarded as excellent. Overall, high values of both statistics (CE_1_ and *d_r_*) showed that the evaluated sigmoid model is suitable for accurately reproducing the shape and trend of consolidation curves. Willmott et al. [64] pointed out that increases and decreases in *CE*_1_ and *d_r_* are monotonic, and both metrics describe the relative extent (in a proportion) to which a set of model predictions is, on average, error-free. However, in some cases, inconsistent result were obtain, and the *CE*_1_ and *d_r_* followed an illogical trend, i.e., higher values for worse fittings. For example, *WE* followed the logical behaviour, i.e., *WE* decreased with decreased *MAE* or *RMSE* values, *CE*_1_ and *d_r_* exhibited the opposite behaviour, i.e., they decreased with improved simulated values as assessed by *MAE* or *RMSE*. Ali and Abustan [65] observed that *CE*_1_ and *d_r_* are more sensitive to observed range/fluctuation. As present analysis shows, despite obtaining very high values of these parameters, which indicates a good fit of the data, the statistical analysis results themselves are not consistent and reliable. A possible cause of the ambiguous results is the absence of externalities (extreme values) in the input data.

As shown in Table 3 and Figure 3 and Figure 4, even when the visually simulated data differ significantly from the observed data, the *CE*_1_ and *d_r_* values are high. Hence, we decided to use one more measure, defining the average tendency of the simulated values to be higher or lower than the observed values. This metric is called percent bias (*PBIAS*) and can be expressed as follows:

(25) $$PBIAS=\left[\frac{\sum_{i=1}^{n}\left(x_{i}-y_{i}\right)*100}{\sum_{i=1}^{n}\left(x_{i}\right)}\right]$$

**Figure 6:**
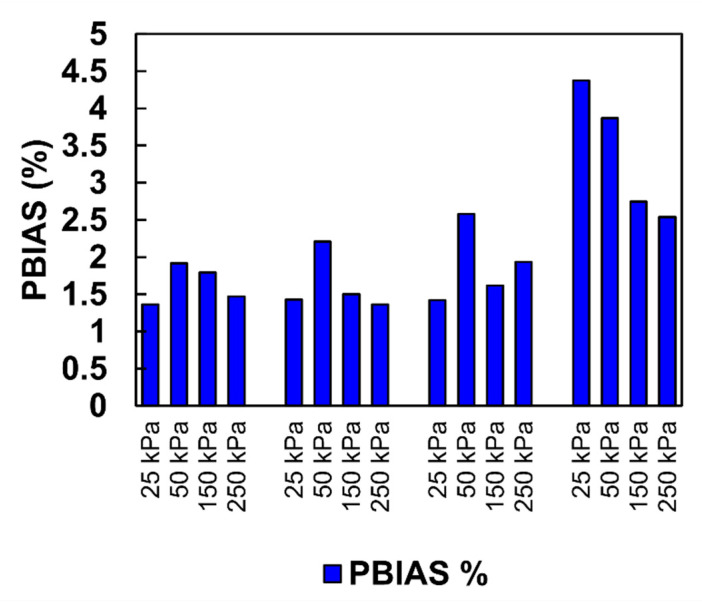
Diagram of PBIAS.

According to Gupta et al. [66], the optimal value of *PBIAS* is 0.0; positive values indicate overestimation bias, whereas negative values indicate model underestimation bias. Figure 6 presents the *PBIAS* values for all considered simulations.

The PBIAS values were positive and ranged from 1.36% to 4.37%. These values indicate that the average magnitude of simulated data was within the very good range, i.e., PBIAS < ±5.

## 6. Conclusions

In summary, various shapes of consolidation curves were investigated through laboratory tests. Four one-dimensional oedometer tests performed on soft, organic, fine-grained soils were used to capture individual behaviour during the consolidation process, i.e., primary and secondary consolidation. The following conclusions were drawn based on the findings of the study:

1. A consolidation curve of various shapes usually represents the laboratory results of the consolidation test. The curve described as an exact mathematical function enables precise determination of any point in its course for any consolidation time. This provides an accurate indication of critical points on the curve, such as tangent to the inflection point, the end time of filtration consolidation, i.e., EOP point or specific time, and the compression necessary to compute the coefficient of consolidation;
2. Optimizing the input data allows for the densification of measurement points, leading to increased accuracy in constitutive modelling when the observed and predicted consolidation courses are compared;
3. In general, the graphical results obtained during calibration indicated adequate model prediction over the range of the average degree of consolidation, and the simulations mostly cover the measurements;
4. Comparisons between observed and predicted data were assessed using various deviation statistics, such as mean error (*E*), root mean square error (*RMSE*), mean absolute error (*MAE*), weighted error (WE), the revised Nash–Sutcliffe efficiency index (*CE*_1_) and the refined index of model performance (*d_r_*). The weighted error (*WE*) was chosen as the optimization target because this normalized metric eliminates the scale effects on the fit between the experimental and simulated results;
5. Although all the statistical measures indicated a perfect match between the experimental and simulated data, some exhibited illogical behaviour, i.e., *CE*_1_ and *d_r_* decreased increased simulated values, as assessed by the *RMSE* or *MAE*. A possible cause of the ambiguous results is the absence of extreme values in the input data. *RMSE* or *MAE* do not provide information about the level or degree of error; therefore, they should be linked to other statistical metrics. According to our statistical analysis, we recommended the use of *RMSE* or *MAE* in combination with *WE* to evaluate the optimization of laboratory data from consolidation studies. Combining these indicators resulted in correct and logical behaviour; *WE* decreased with decreased *RMSE* or *MAE* values;
6. All findings based on statistical assessments demonstrate that the evaluated sigmoid model is efficient and applicable for accurate reproduction of various shapes of laboratory consolidation curves and is therefore a valuable tool for numerical analysis.

## Figures and Tables

**Table 1:** Physical parameters of intact soft organic soils utilised in the present study.

		Particle Size				Atterberg Limits				
Soil Type	Sample	Sand (%)	Silt (%)	Clay (%)	Natural Water Content (%)	Liquid Limit (%)	Plastic Limit (%)	Plasticity Index (%)	Organic Content (%)	Specific Gravity (-)
Organic silty clay	O1	13	68	19	60.30	82.96	33.33	49.63	7.00	2.56
Organic clayey silt	O2	2	56	42	84.00	109.50	54.22	55.28	11.30	2.62
Organic clayey silt	O3	23	60	17	55.22	66.11	32.80	33.31	5.00	2.61
Organic clayey silt	O4	18	61	21	51.75	75.22	37.50	37.72	6.80	2.63

**Table 2:** Optimal sets of parameters for Mexico clay and calculated deviation statistics.

*a*_1_	*a*_2_	*x*_0_	*n*	*R* ^2^	*E*	*RMSE*	*MAE*	*WE*	*CE*_1_	*d_r_*
[-]	[-]	[-]	[-]	[-]	[-]	[-]	[-]	[-]	[-]	[-]
**Type I**										
0.012807	0.000372	9982.806	0.991025	0.998	−0.0017	0.0110	0.0080	0.0211	0.968	0.991
**Type II**										
0.036131	0.000902	648.8058	0.79307	0.981	0.448297	0.0443	0.0094	0.119	0.826	0.926
**Type III**										
0.002755	0.000663	22.80575	0.670025	0.999	0.006929	0.0084	0.0069	0.027	0.931	0.982

**Table 3:** Optimal sets of parameters for soft organic soils and calculated deviation statistics.

*σ*	*A*1	*A*2	*x*_0_	*p*	*R* ^2^	*E*	*RMSE*	*MAE*	*WE*	*CE*_1_	*d_r_*
[kPa]	[-]	[-]	[-]	[-]	[-]	[-]	[-]	[-]	[-]	[-]	[-]
**Sample O1**											
25	0.03087	0.000262	2999.806	1.004025	0.99914	0.0009	0.01133	0.00699	0.017	0.977	0.997
50	0.02807	0.000472	2082.806	0.970025	0.99875	−0.0036	0.01275	0.00889	0.019	0.989	0.996
150	0.04180	0.000252	1282.806	0.970025	0.99758	0.0027	0.01784	0.00960	0.021	0.942	0.984
250	0.02280	0.000222	1882.806	0.985025	0.99866	0.0009	0.01406	0.00819	0.015	0.983	0.988
**Sample O2**											
25	0.02194	0.000632	5952.806	1.220703	0.99925	0.0012	0.01059	0.00720	0.022	0.976	0.986
50	0.00689	1.82 × 10^−5^	12992.81	0.848267	0.99918	−0.0003	0.01052	0.00824	0.025	0.984	0.998
150	0.01160	0.000272	5382.806	0.999025	0.99945	0.0048	0.01033	0.00688	0.016	0.920	0.976
250	0.01010	0.000202	5312.806	0.999025	0.99924	−0.0002	0.01002	0.00716	0.025	0.986	0.998
**Sample O3**											
25	0.02797	0.000568	3982.806	1.180054	0.99920	0.0036	0.01191	0.00722	0.020	0.978	0.981
50	0.02807	0.000524	1099.806	0.870025	0.99727	−0.0011	0.01776	0.01127	0.022	0.976	0.990
150	0.04780	0.000292	1222.806	0.970025	0.99746	0.0027	0.01820	0.00929	0.017	0.965	0.983
250	0.02790	0.000195	1492.862	0.999925	0.99814	0.0002	0.01564	0.01083	0.031	0.978	0.996
**Sample O4**											
25	0.05423	0.000182	368.8058	0.89707	0.99465	0.0003	0.02925	0.02134	0.061	0.984	0.997
50	0.05413	0.000112	320.6058	0.85707	0.99585	0.0047	0.02846	0.02003	0.056	0.989	0.998
150	0.07013	0.000452	141.9958	0.77207	0.99464	0.0032	0.02224	0.01157	0.019	0.961	0.986
250	0.03673	0.000202	448.8058	0.79207	0.99671	−0.0004	0.01872	0.01217	0.030	0.982	0.997

## Data Availability

Not applicable.
